# Supplementary material for: The impact of Arctic sea ice loss on mid-Holocene climate
Source: Nat Commun. 2018 Nov 1;9:4571. doi: 10.1038/s41467-018-07068-2 (PMC6212424; doi:10.1038/s41467-018-07068-2)
Supplement: Supplementary file 1 — Supplementary Information [file 41467_2018_7068_MOESM1_ESM.pdf]

Supplementary information:

The impact of Arctic sea ice loss on mid-Holocene climate

Park et al.

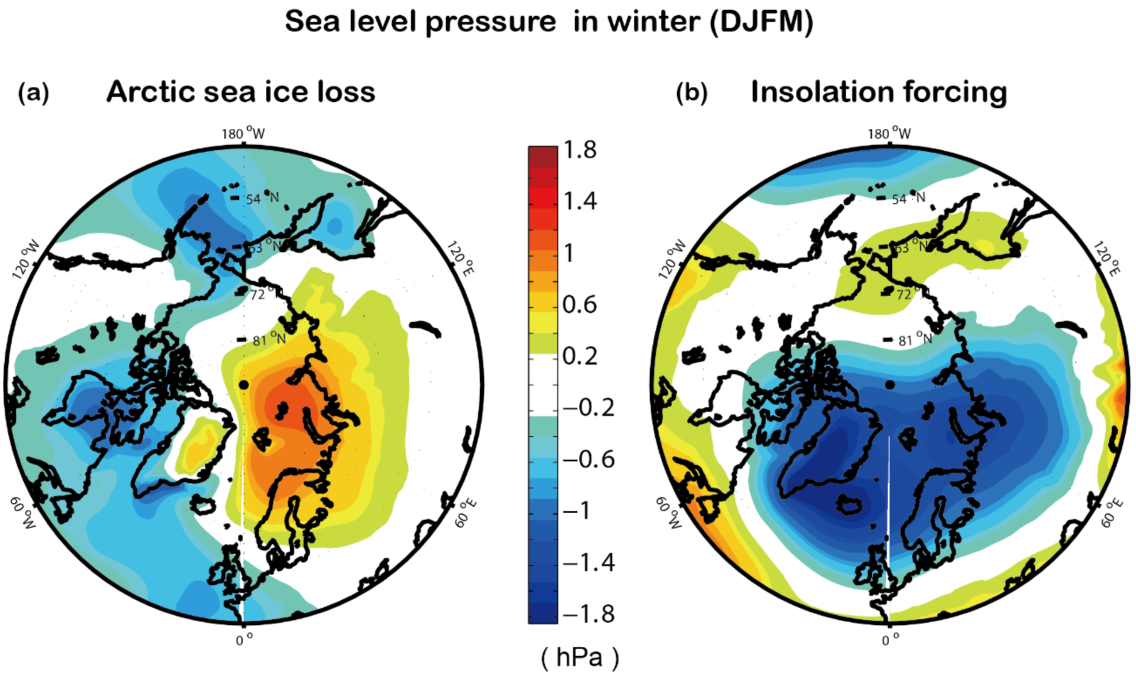

Supplementary Figure 1: Sea level pressure responses to **(a)** Arctic sea ice loss and **(b)** insolation forcing in winter (December–March).

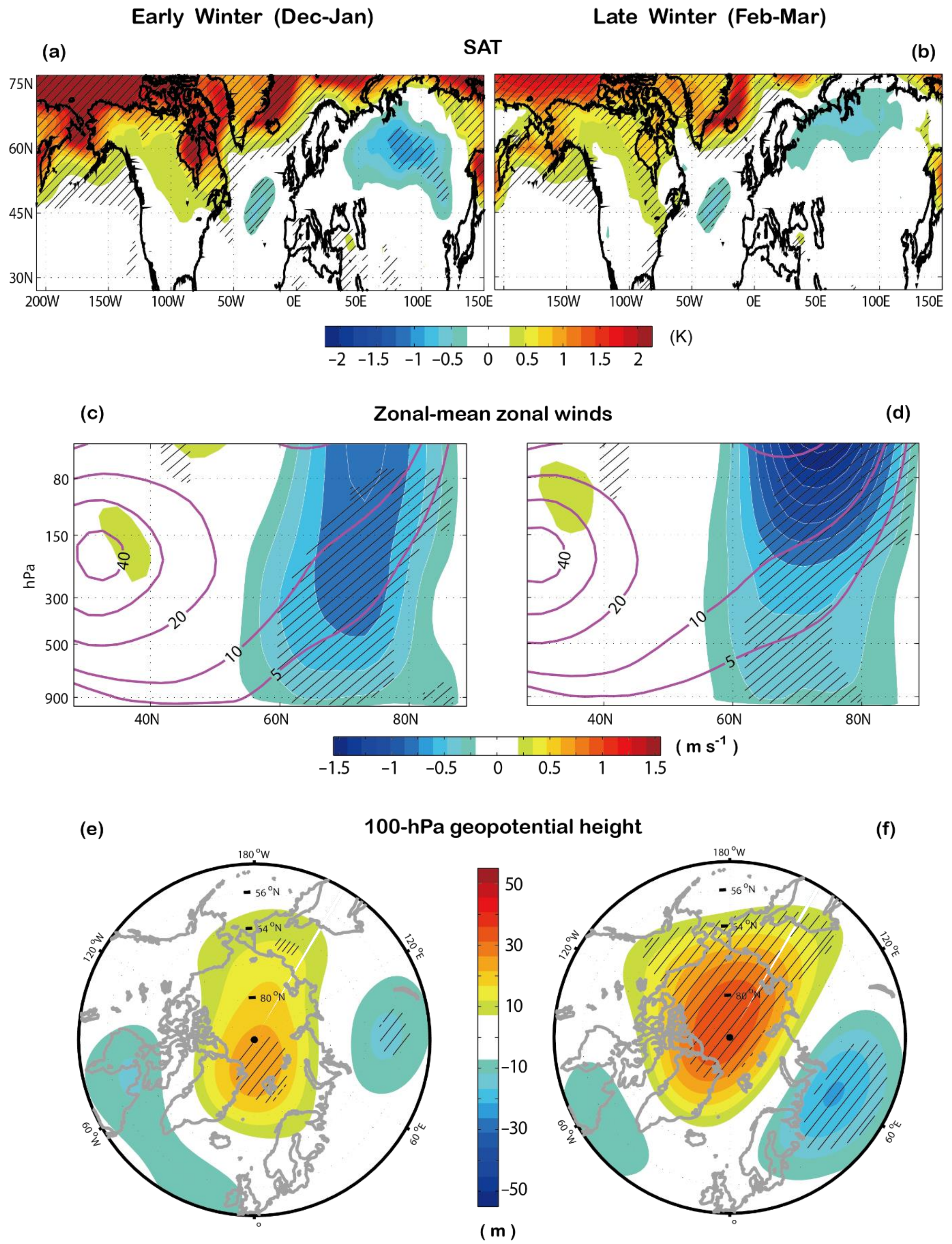

Supplementary Figure 2: **(a, b)** Surface air temperature (K), **(c, d)** zonal-mean zonal winds (m s^{-1}) and **(e, f)** 100-hPa geopotential height (m) responses to Arctic sea ice loss during **(**left

column: **a, c, e)** early winter (December – January) and **(**right column: **b, d, f)** late winter (February – March). In (**c, d**), purple lines are climatological-mean zonal-mean zonal winds from the mid-Holocene with pre-industrial sea ice simulation. For all plots, statistically significant values ($p < 0.05$) are hatched.

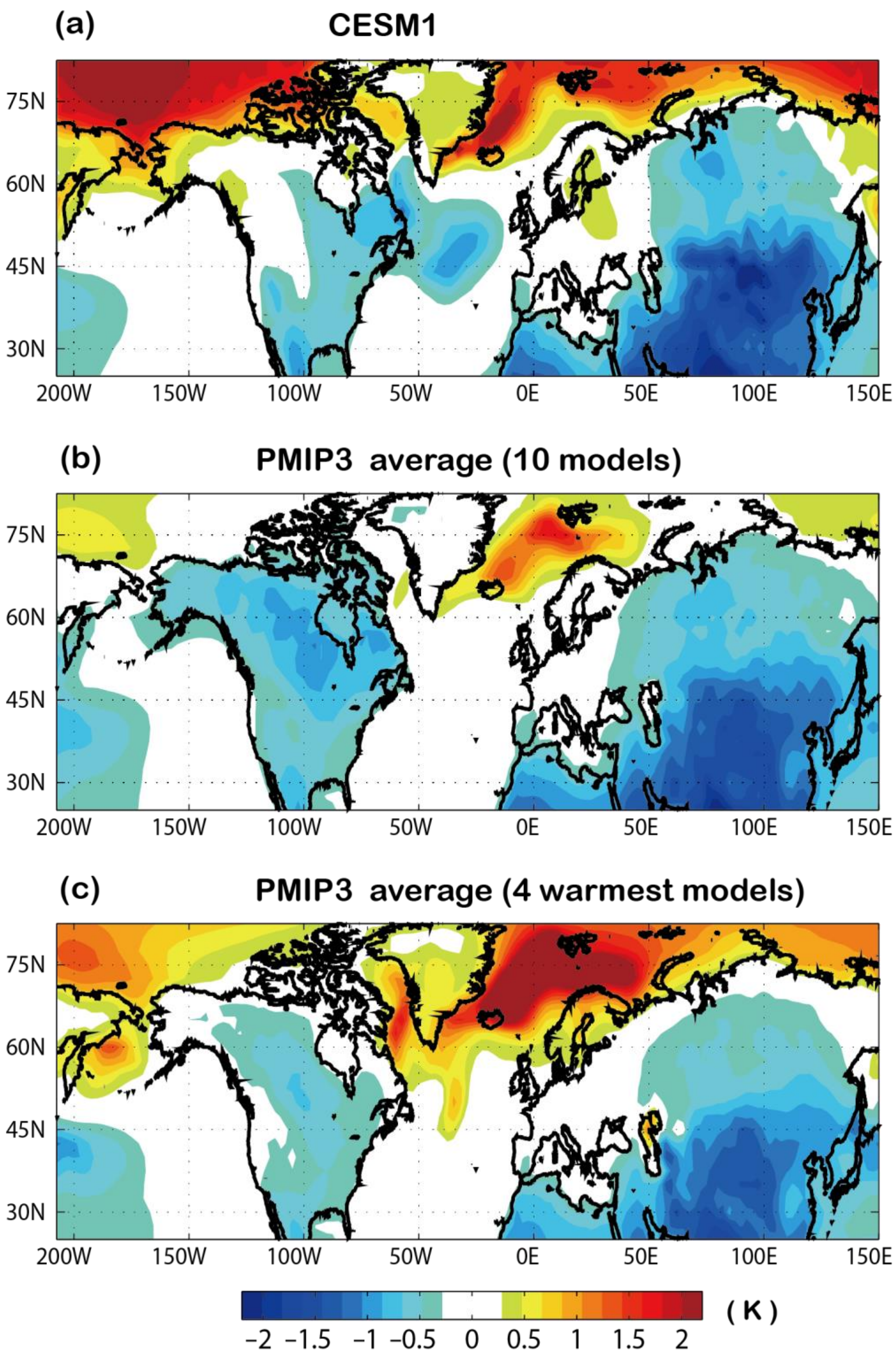

Supplementary Figure 3: The wintertime (December–March) SAT anomalies in the mid-Holocene simulation (differences between the mid-Holocene and pre-industrial simulations) from **(a)** CESM1, **(b)** the PMIP3 multi-model average (10 models) and **(c)** PMIP3 average of 4 warmest models in the Arctic.

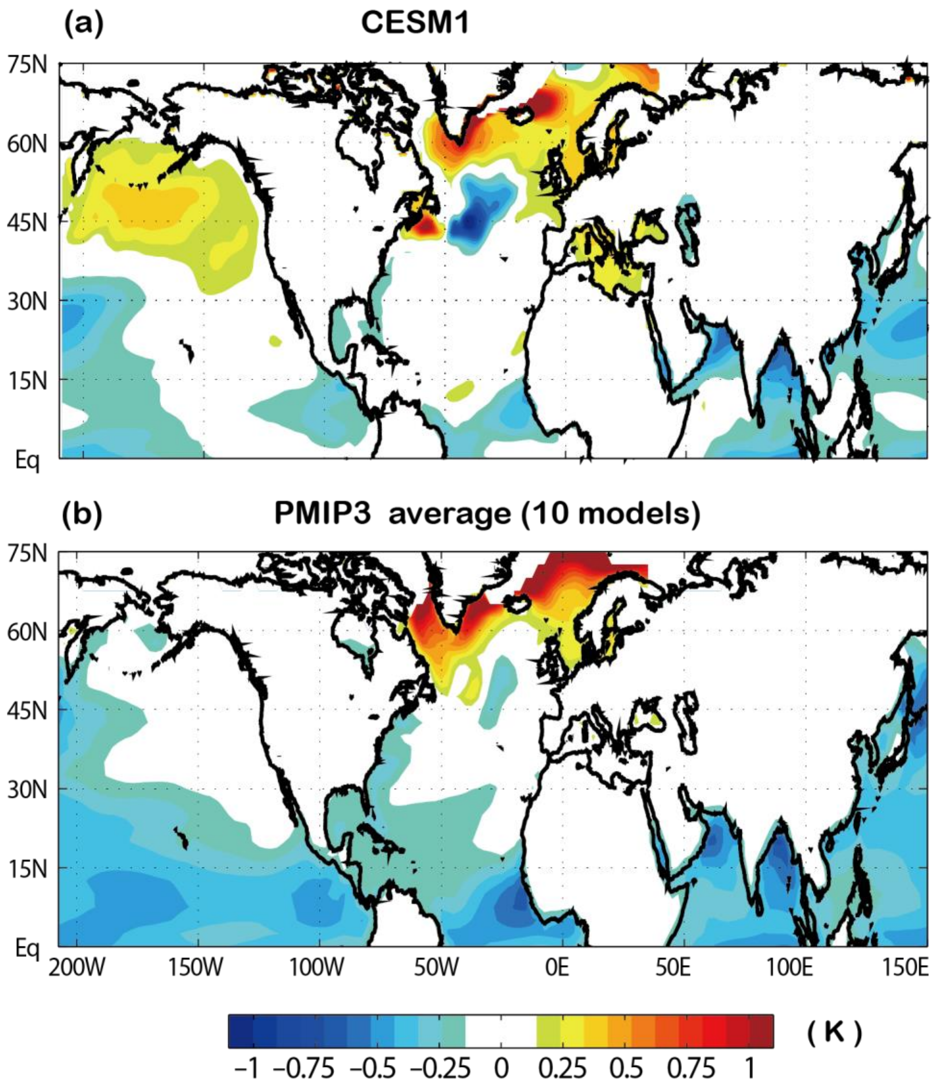

Supplementary Figure 4: The annual-mean SST anomalies in the mid-Holocene simulation (differences between the mid-Holocene and pre-industrial simulations) from **(a)** CESM1 and **(b)** the PMIP3 multi-model average (10 models).

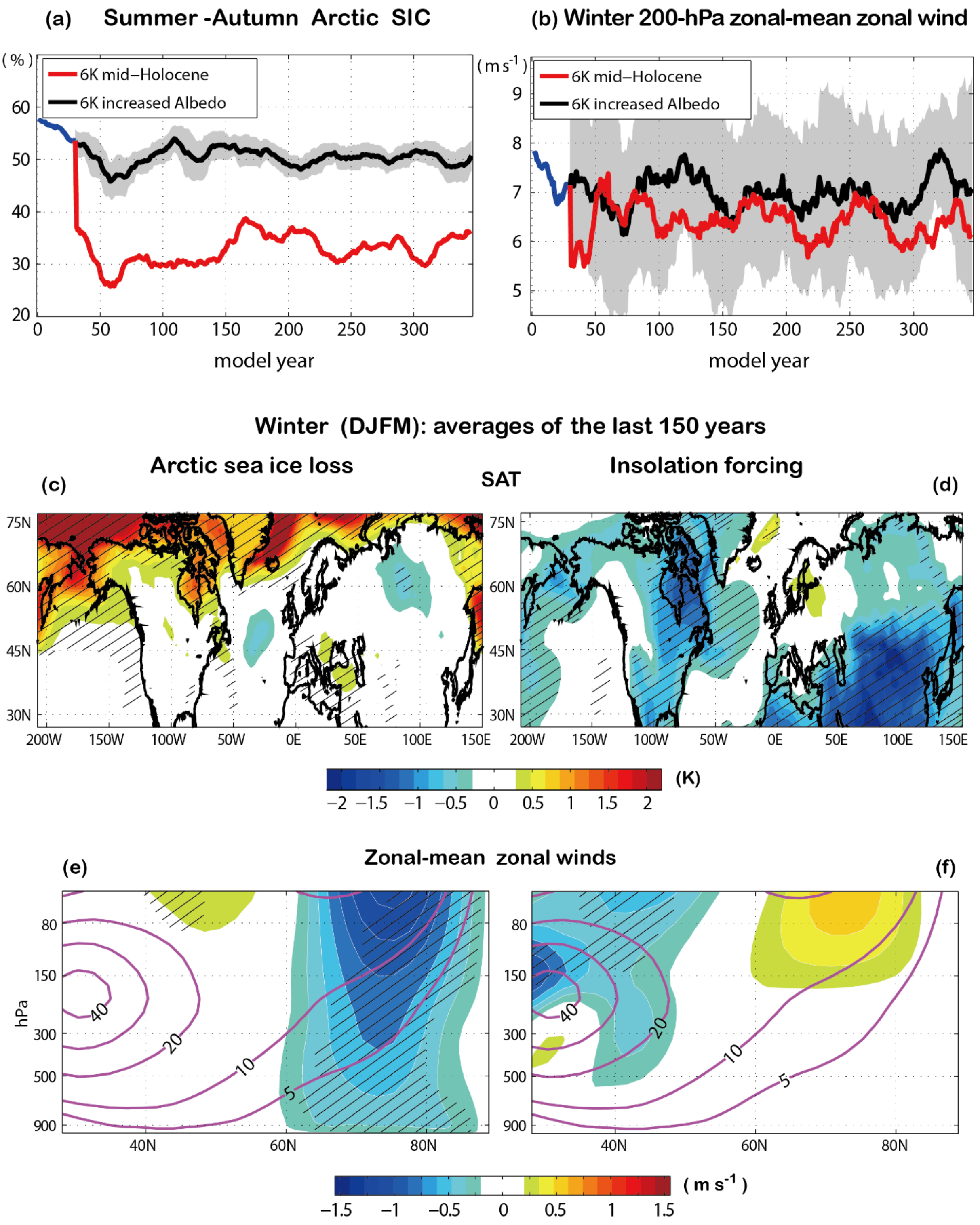

Supplementary Figure 5: The 20-year running averages of **(a)** Arctic summer (July–November) sea ice concentration (%) and **(b)** the wintertime (December–March) 200-hPa zonal-mean zonal wind speed (m s-1) averaged from 65°N to 80°N, for the mid-Holocene (red),

the mid-Holocene with pre-industrial sea ice (black), and the pre-industrial control (blue) simulations. The gray shadings indicate the range of 1 standard deviation from the mean of the mid-Holocene with pre-industrial sea ice (black) simulations. The wintertime (December–March) responses of **(c, d)** surface air temperature (K) and **(e, f)** zonal-mean zonal winds (m s^{-1}) to mid-Holocene Arctic sea ice loss **(c, e)** and insolation forcing **(d, f)**, averaged over the last 150 years. Supplmentary Fig. **5(c, d, e, f)** are same as Fig. **3(a, b, c, d)**, except that the averaging periods between these two plots are different (last 150 years vs. last 265 years).

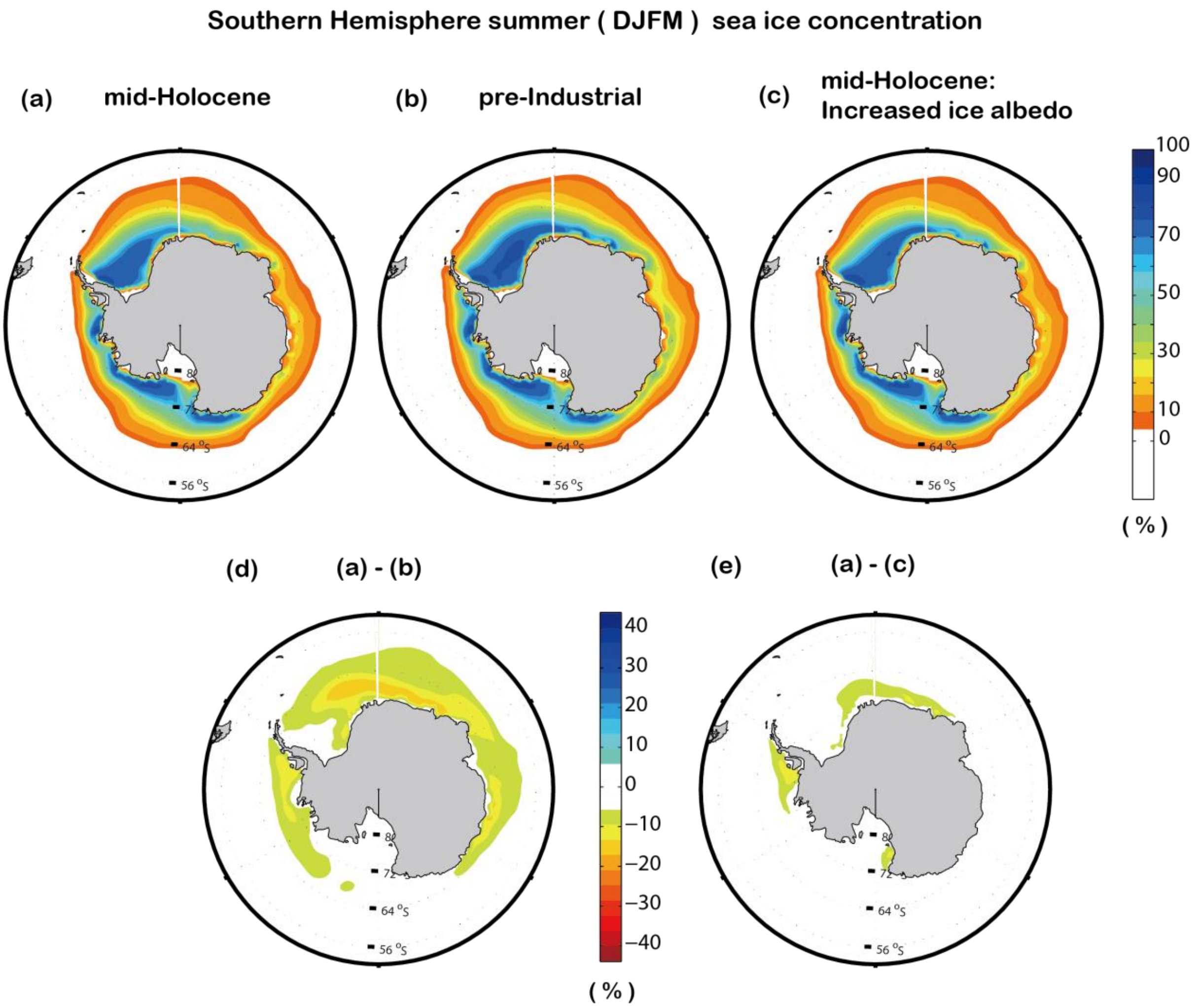

Supp. Figure 6: Antarctic sea ice concentration (%) from **(a)** the mid-Holocene simulation, **(b)** the pre-industrial simulation and **(c)** the mid-Holocene simulation with increased ice albedo, during southern hemisphere summer (DJFM). Sea ice concentration differences **(d)** between mid-Holocene and pre-industrial simulations, i.e. **(a)**–**(b)**, and **(e)** between mid-Holocene and mid-Holocene with increased ice albedo simulations, i.e. **(a)**–**(c)**.
